# Urinary gonadotropin assay on 24-h collections as a tool to detect early central puberty onset in girls: determination of predictive thresholds

**DOI:** 10.1093/humrep/deae055

**Published:** 2024-03-21

**Authors:** Clément Janot, Pauline Perrin, Véronique Raverot, Patricia Bretones, René Ecochard, Sarah Malburet-Testori, Marc Nicolino, Zoé Robert, Florence Roucher-Boulez, Carine Villanueva, Kevin Perge, Ingrid Plotton

**Affiliations:** Service de Biochimie et Biologie moléculaire, Centre de Biologie et de Pathologie Est, Hospices Civils de Lyon, LBMMS, Bron Cedex, France; Faculté de Médecine Lyon Est, Université Claude Bernard Lyon 1, Lyon, France; StemGamE Platform group, UMR INSERM 1208 SBRI, Bron, France; Service de Biochimie et Biologie moléculaire, Centre de Biologie et de Pathologie Est, Hospices Civils de Lyon, LBMMS, Bron Cedex, France; Service de Biochimie et Biologie moléculaire, Centre de Biologie et de Pathologie Est, Hospices Civils de Lyon, LBMMS, Bron Cedex, France; Groupement Hospitalier Est, Service d’Endocrinologie pédiatrique, Hospices Civils de Lyon, Bron Cedex, France; Laboratoire Biostatistique Santé, UMR CNRS 5558 UCBL, Lyon, France; Service de Biochimie et Biologie moléculaire, Centre de Biologie et de Pathologie Est, Hospices Civils de Lyon, LBMMS, Bron Cedex, France; Faculté de Médecine Lyon Est, Université Claude Bernard Lyon 1, Lyon, France; Groupement Hospitalier Est, Service d’Endocrinologie pédiatrique, Hospices Civils de Lyon, Bron Cedex, France; Service de Biochimie et Biologie moléculaire, Centre de Biologie et de Pathologie Est, Hospices Civils de Lyon, LBMMS, Bron Cedex, France; Service de Biochimie et Biologie moléculaire, Centre de Biologie et de Pathologie Est, Hospices Civils de Lyon, LBMMS, Bron Cedex, France; Faculté de Médecine Lyon Est, Université Claude Bernard Lyon 1, Lyon, France; StemGamE Platform group, UMR INSERM 1208 SBRI, Bron, France; Faculté de Médecine Lyon Est, Université Claude Bernard Lyon 1, Lyon, France; Groupement Hospitalier Est, Service d’Endocrinologie pédiatrique, Hospices Civils de Lyon, Bron Cedex, France; Faculté de Médecine Lyon Est, Université Claude Bernard Lyon 1, Lyon, France; Groupement Hospitalier Est, Service d’Endocrinologie pédiatrique, Hospices Civils de Lyon, Bron Cedex, France; Service de Biochimie et Biologie moléculaire, Centre de Biologie et de Pathologie Est, Hospices Civils de Lyon, LBMMS, Bron Cedex, France; Faculté de Médecine Lyon Est, Université Claude Bernard Lyon 1, Lyon, France; StemGamE Platform group, UMR INSERM 1208 SBRI, Bron, France

**Keywords:** precocious puberty, gonadotropins, hormones, children, urine

## Abstract

**STUDY QUESTION:**

Is the 24-h urinary gonadotropin assay an effective diagnostic tool in central precocious puberty (CPP) in girls?

**SUMMARY ANSWER:**

This study is the first to provide 24-h urinary gonadotropin assay data, using an electrochemiluminescent immunoassay (CMIA), and to report its usefulness as a tool for the diagnosis of CPP.

**WHAT IS KNOWN ALREADY:**

Data about the GnRH test in the diagnosis of CPP are variable and there is no consensus regarding its interpretation. The measurement of FSH and LH in urines was previously reported to be an alternative biological tool.

**STUDY DESIGN, SIZE, DURATION:**

This is a retrospective two-cohort study, involving a setting and a validation cohort. A total of 516 girls, included between October 2012 and July 2015, and 632 urinary collections were analyzed in the setting cohort. In the validation cohort, 39 girls were included between January 2021 and May 2023, and 49 urinary collections were analyzed.

**PARTICIPANTS/MATERIALS, SETTING, METHODS:**

This study included girls who consulted for an investigation of disturbed growth rate or a clinical suspicion of puberty onset in different medical centres across France (setting cohort). Girls with a suspicion of precocious puberty onset were addressed at the expert centre of paediatric endocrinology of the Groupement Hospitalier Lyon Est (validation cohort). Pelvic ultrasonography was performed and enabled their classification according to clinical and morphologic changes criteria (prepubertal or pubertal groups). The parents collected 24-h urine samples (u_24_) according to standardized instructions. FSH and LH (urinary or plasmatic) were measured using a current and automated CMIA.

**MAIN RESULTS AND THE ROLE OF CHANCE:**

The area under the ROC curves for CPP prediction was 0.709 for u_24_FSH (*P* < 0.001), 0.767 for u_24_LH (*P* < 0.001), and 0.753 for the u_24_LH/u_24_FSH ratio (*P* < 0.001). We retained all possible combinations of the four thresholds in the validation cohort (u_24_FSH = 1.1 or 2.0 IU/24 h; u_24_LH = 0.035 or 0.08 IU/24 h). The combination of u_24_FSH > 1.1 IU/24 h and u_24_LH > 0.08 IU/24 h had a positive PV of 85.7% and a negative PV of 94.3%, a sensitivity of 85.7% and a specificity of 94.3%, for classifying prepubertal and pubertal girls in this cohort.

**LIMITATIONS, REASONS FOR CAUTION:**

This is a retrospective study, in which a margin of error remains due to the inherent uncertainty regarding the clinical assessment of pubertal onset. It must be considered that the thresholds can only apply to the used reagents; measurements without extractions using other reagents are likely to show important heterogeneity.

**WIDER IMPLICATIONS OF THE FINDINGS:**

The assay performed herein is a simple, non-invasive, and analytically robust technique meeting the criteria for an alternative to the GnRH test which could be used to supplement its lack of sensitivity.

**STUDY FUNDING/COMPETING INTEREST(S):**

No specific funding was used. All authors declared no conflict of interest.

**TRIAL REGISTRATION NUMBER:**

In-house #23-5214 registered study.

## Introduction

Central precocious puberty (CPP) is caused by the early activation of the hypothalamic–pituitary–gonadal (HPG) axis. The prevalence was estimated to be from 1 in 5000 to 1 in 10 000 children, having a 10:1 girl:boy ratio (Carel and Léger, 2008; Maione *et al.*, 2021). The diagnosis is of crucial importance, as a search for the etiological causes, using cerebral MRI, could lead to the identification of lesions of the central nervous system, reported in <1–3% of 6–8 years old children, and up to 6–25% of <6 years old cases (Cantas-Orsdemir *et al.*, 2018). In girls, precocious puberty onset is clinically defined by the development of secondary sexual characteristics before 8 years of age (Eckert-Lind *et al.*, 2020; Roberts and Kaiser, 2020); breast development is the first clinical sign leading to its suspicion. As clinical examination of breast development may be difficult to confirm, a hormonal assessment is essential before introducing treatment in the case of CPP. To confirm the onset of CPP, the current guidelines (Carel *et al.*, 2009; Bangalore Krishna *et al.*, 2019) recommend the measurement of plasma LH using sensitive chemiluminescent or electrochemiluminescent immunoassay (CMIA or ECLIA) reagents, before or after a GnRH stimulation test (Kastin *et al.*, 1972a,b). However, their diagnostic performances are flawed due to a significant overlap in hormone levels between the prepubertal stage, premature thelarche, and central puberty onset (Carel *et al.*, 2009; Bangalore Krishna *et al.*, 2019). In girls, a pelvic ultrasound can reveal morphologic evolution of the internal genitalia, and is highly specific and non-invasive, but it lacks of sensitivity (De Vries *et al.*, 2006; Sathasivam *et al.*, 2011).

The GnRH test is widely used by practitioners, although there are different well-defined hormone levels thresholds after stimulation (Carel and Léger, 2008; Brito *et al.*, 2016). Nevertheless, an LH peak >5 IU/l associated with an LH/FSH peak ratio >0.66 is commonly adopted by the paediatrician community. Previous studies have reported variable protocols, using different times for blood sampling, and different analytical methods for measurements of FSH and LH (Kandemir *et al.*, 2011; Brito *et al.*, 2016; Ab Rahim *et al.*, 2020; Huynh *et al.*, 2022). The GnRH stimulation test is also invasive and stressful, involving an intravenous injection and one to six repeated samples under supervision, usually requiring day-hospitalization. The measurement of FSH and LH in urine samples was previously reported to be a useful biological tool to detect the pubertal increase of gonadotropin secretion (Buckler and Clayton, 1970; Rifkind *et al.*, 1970; Chipman *et al.*, 1981). Several studies have reported interesting results regarding the usefulness of first-morning voided (FMV) or random non-timed urines to detect puberty onset with good sensitivity in girls (McNeilly *et al.*, 2012; Zung *et al.*, 2014; Demir *et al.*, 2016, 2021; Lucaccioni *et al.*, 2016; Kolby *et al.*, 2017; Shim *et al.*, 2019; Yüce *et al.*, 2020; Lee *et al.*, 2021; Zhan *et al.*, 2021). The collection of 24-h urine samples provides the complete nychthemeral activity of the HPG axis and the data correlates well with that from morning urine samples (Maesaka *et al.*, 1990). However, few data have been reported about 24-h urinary collections for precocious puberty, except where radioimmunological techniques have been used (Morel *et al.*, 1985).

The aim of this study was to assess 24-h urinary gonadotropin measurements as a tool for girls presenting with a clinical suspicion of CPP, by establishing and then validating the decision thresholds of FSH (u_24_FSH) and LH (u_24_LH).

## Materials and methods

### Study design and population

This is a retrospective two-cohort study, involving a setting cohort and a validation cohort.

For the setting cohort, we identified girls who consulted for an investigation of disturbed growth rate or a clinical suspicion of puberty onset in different medical centres across the Auvergne–Rhône–Alpes region and beyond (France) between October 2012 and July 2015. We included girls for whom (i) the Tanner pubertal stage for breast development was determined at the time of the consultation, prior to urine collection; and (ii) the 24-h urinary collections were addressed for the u_24_FSH and u_24_LH assays to the Hormonology unit of Biochemistry and Molecular Biology department of Groupement Hospitalier Est (Hospices civils de Lyon, France). We excluded girls: (i) presenting with endocrine disease which interferes with the onset of puberty (congenital adrenal hyperplasia, peripheral puberty onset, gonadal diseases, growth hormone deficiency, thyroid hormone deficiency, syndromic pathologies, etc.); (ii) younger than 4 years old, in order to eliminate the ‘minipuberty’ due to transient late activation of the HPG axis; (iii) with urinary collections under 400 ml or without available collection volumes; or (iv) with doubtful or inconclusive determination of Tanner stages.

The Tanner classification of breast development followed the detailed clinical description previously provided and used worldwide (Marshall and Tanner, 1969; Morris and Udry, 1980; Carel and Léger, 2008). In case of unilateral right or left breast classified as S2 (and the other S1), the patient was considered S2.

In this cohort, the urine–plasma correlations as well as the distribution of urinary values regarding Tanner stages were analyzed. We established thresholds that allowed us to characterize the puberty onset using 24-h urinary gonadotropins by using S1 and S2 values. We included GnRH stimulation tests when they were performed at the time of consultation. This test consisted in an intravenous injection of 2.5 μg/kg or up to 100 μg of gonadorelin acetate. Blood samples were collected prior to the injection (T0), then 30, 60, and 90 min after injection: peak values of FSH and LH during the test were measured. The stimulation test was considered positive when the LH peak was higher than 5 IU/l, or when the LH/FSH peak ratio was higher than 0.66. It was decided to consider the peak of the LH or that of the ratio so as not to artificially reduce the sensitivity of the test.

For the validation cohort, we identified girls with a suspicion of precocious puberty onset consulted with fifteen practitioners in the Department of Pediatric Endocrinology and Metabolic Diseases of the Groupement Hospitalier Est between January 2021 and May 2023. The suspicion of precocious puberty was defined as girls for whom the first clinical signs occurred before 8 years of age, considering the delay between puberty onset and consultation. The inclusion criteria of this validation cohort were the same as those of the setting cohort. Were excluded girls: (i) with doubtful or inconclusive Tanner stages (‘unilateral’ (right or left) S2 were considered S2); (ii) with urinary collections under 600 ml; (iii) older than 8 years old at the time of clinical onset; (iv) with peripheral precocious puberty; or (v) ongoing puberty blocking treatment. As the aim in this study was to differentiate pre-pubertal from pubertal patients, girls with breast development stage ≥S2 were merged in a S2 group. Pelvic ultrasonography (US) was performed in all girls in the S2 group in our centre. This enabled their classification into two groups: (i) pubertal S2US+ with girls who had patterns of morphologic enlargement of the uterus and ovaries due to estrogen effects, according to previously reported criteria (Table 1, derived from Stranzinger and Strouse, 2008; Asavoaie, 2014), and (ii) S2US− were girls who had no such patterns. The S1 and S2US− girls constituted the prepubertal group.

**Table 1.** Ultrasonography criteria for HPG axis activation.

	Prepubertal (US−)	Pubertal (US+)
Uterus		
Shape	tubular shape	pear shape
Volume (ml)	<1.6	>1.6
Fundus/cervix size ratio	≤1:1	>1:1
Endometrial line	absent	Visible
Endometrial stripe	hypoechoic	Echogenic
Ovaries		
Volume (ml)	<2	≥2

For this cohort, the aim was to validate the previously established u_24_FSH and u_24_LH thresholds according to the breast Tanner stages (same criteria than previously) and the US data in the prepubertal and pubertal groups. Urinary gonadotropin assays were performed and obtained as part of routine clinical practice. The clinical progression of puberty at a second consultation was included if available, in order to study the concordance with urinary findings. Evolution of breast size was used as clinical criterion to on-course progressive precocious puberty.

### Urinary collections

The parents collected the 24-h urine according to standardized instructions: patients were asked to discard the last evening’s voiding before going to bed and note the time. During the following 24 h, all subsequent urine samples were collected, in a clean, dry, and empty canteen without additives, until the time recorded the previous day. The urine was homogenized at the end of the collection. The total volume was measured and a sample of the homogenate was placed in a tube without additives. The assay was performed on a non-frozen aliquot less than a week after the end of the collection.

### FSH and LH measurements

FSH and LH (urinary or plasmatic) were measured by a current and automated CMIA using Architect i2000 analyzer from Abbott Laboratories^®^ (IL 60064, USA; FSH reagent ref. 7K75; LH reagent ref. 2P40). No modification was carried out to the assays, whether they were used for urine or plasma. Both assays are calibrated against the international reference materials from World Health Organization (WHO), 92/510 and 80/552, respectively, for FSH and LH. Assays on urine were performed on samples stored at 4 °C that were never frozen, free of any previous treatment or extraction, rapidly vortexed and centrifuged just before measurement. The analytical method applied to urine in this work was validated in the hormonology laboratory, and is highly correlated with the immunoradiometric assay (IRMA) technique, notably on extraction-free urine (Supplementary Fig. S1 and Table S1). Plasma gonadotropins were collected in ethylene diamine tetra-acetic acid tubes. Intra-assay coefficients of variation were <4.2% for plasma FSH, <3.6% for plasma LH (determined by Abbott Laboratories^®^), <4.1% for urinary FSH and <6.5% for urinary LH (determined by in-house tests on urinary samples). Inter-assay coefficients of variation were <2.8% for plasma FSH, <2.7% for plasma LH, <3.3% for urinary FSH, and <4.5% for urinary LH (determined by the long-term use of Technopath^®^ Multichem U and Multichem IA plus Quality Controls; from Technopath Clinical Diagnostics, Ballina, Ireland). Urinary assays were related to the volume of diuresis and expressed as daily excretion (u_24_FSH and u_24_LH, in IU/24h).

### Statistical analyses

The median of u_24_FSH and u_24_LH were used for distribution analysis and statistical comparison using the non-parametric Kruskal–Wallis test. *Post hoc* Dunn’s test was used for multiple comparisons. Mann–Whitney test was used for comparisons between GnRH+ and GnRH− groups. Relationships between u_24_FSH, u_24_LH and plasmatic FSH and LH were analyzed using Spearman correlation. Thresholds establishment used the Youden index (YI) (*Y* = sensitivity + specificity − 1). An alpha risk of error was set at 5% for all tests and confidence intervals. All statistic tests, receiver operating characteristic (ROC) and precision–recall curves and threshold establishment were performed using the GraphPad Prism^®^ version 6.01 (GraphPad Software, Boston, MA, USA).

### Ethical approval

The present study protocol was approved by the ethics committee of our institution (23-5214). Medical and biological personal data in this research were studied anonymously. Their use followed good practices and the principles of the Declaration of Helsinki as well as the European legislation on personal data protection (MR-004 GDPR standards).

## Results

### Setting cohort

**Figure 1.**
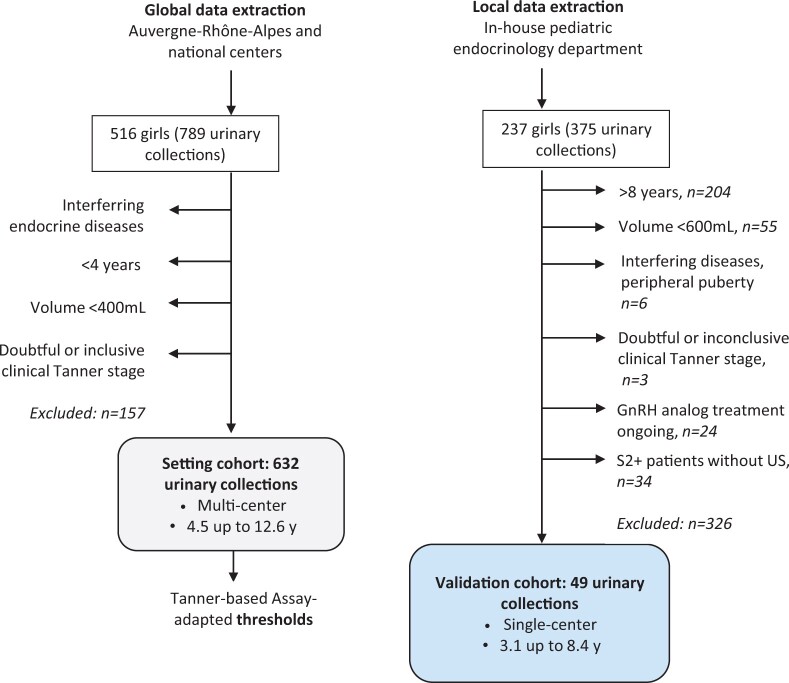
**Flowchart of inclusion and exclusion steps of setting and validation cohorts.** GnRH, gonadotropin-releasing hormone; S2+, Breast Tanner stage or more; US, ultrasonography.

**Figure 2.**
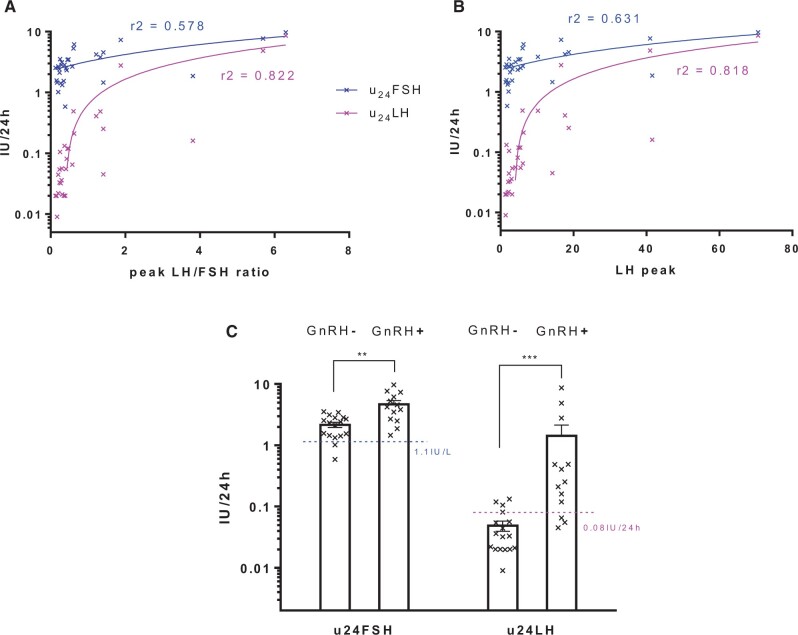
**Correlations between 24-h urinary gonadotropins and GnRH tests.** (**A** and **B**) Correlation (*r* = Spearman coefficient) with LH peak and LH/FSH peak ration after GnRH test in 30 urinary collection (23 patients). (**C**) Distribution of u_24_FSH and u_24_LH according to GnRH test response. Blue- and purple-hashed lines respectively correspond with u_24_FSH and u_24_LH thresholds used hereafter (**P = 0.001; ***P < 0.0001; Mann–Whitney tests). FSH, follicle-stimulating hormone; GnRH, gonadotropin-releasing hormone; LH, luteinizing hormone.

A total of 516 girls with 789 urinary collections were initially selected. After exclusions, 632 urinary collections were finally analyzed in this cohort (Fig. 1). Among them, 23 patients underwent the GnRH stimulation test (providing 30 stimulation and collections tests), and 159 patients had blood collection for basal plasma FSH and LH assays. There were 17 girls who had a negative test (GnRH−) and 13 who had a positive test (GnRH+). Interestingly, u_24_FSH and u_24_LH were correlated with both the LH peak and the LH/FSH peak ratio (Fig. 2A and B). However, the FSH peak alone was not correlated with either u_24_FSH or u_24_LH. U_24_FSH and u_24_LH were both higher in GnRH+ patients compared with GnRH− patients (*P* = 0.001 and *P* < 0.001, respectively). Among the 13 GnRH+ girls, four had an u_24_LH and five had an u_24_FSH readings within the range of the GnRH− girls (Fig. 2C). Basal plasma FSH and LH were respectively correlated with u_24_FSH (*r*^2^ = 0.784, *P* < 0.001) and u_24_LH (*r*^2^ = 0.715, *P* < 0.001).

**Figure 3.**
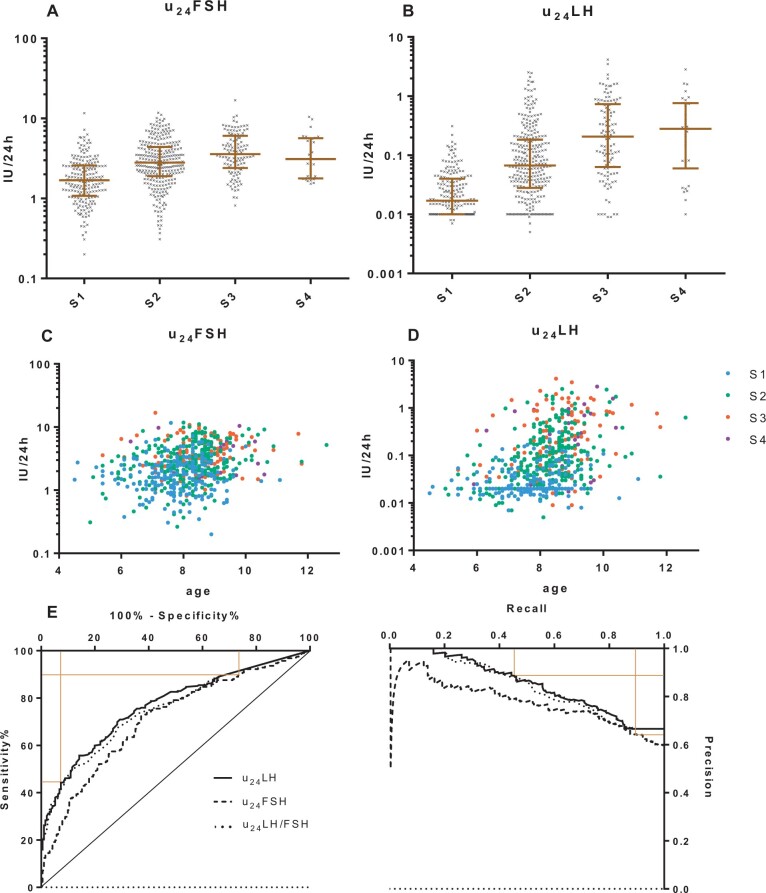
**Distributions of values in the setting cohort and receiver operating characteristic (ROC) curves.** (**A** and **B**) Distribution of values in log-10 scale per Tanner breast stages. Undetectable u_24_LH are represented as aligned dots on the *y* = LLOD line, namely 0.01 IU (B). (**C** and **D**) Urinary gonadotropins according to Tanner breast stage and age. (**E**) Receiver operating (sensitivity and specificity) and Precision–Recall (precision=predictive value; recall = sensitivity) curves. Horizontal and vertical lines point finally validated thresholds. FSH, follicle-stimulating hormone; LH, luteinizing hormone; LLOD, low limit of detection.

The number of patients and samples as well as the ages per Tanner stage groups are shown in Table 2. The distribution of u_24_FSH (Fig. 3A–C) showed a wide range of values for Tanner stage from 1 to 4. Although there was a significant difference between the four stages, and the S2 mean value was higher than that of S1 (*P* < 0.001), the overlap of the S1 and S2 intervals was important. One outlier point was identified in the S1 group (u_24_FSH = 11.62 IU/24 h). The distribution of u_24_LH (Fig. 3B–D) showed a narrow range of values in S1 girls (up to 0.31 IU/24 h) but was higher in S2 girls (up to 2.51 IU/24 h). There was a significant difference according to Tanner stages (*P* < 0.001). The proportion of undetectable u_24_LH (less than the lower limit of detection, LLOD) was the highest in S1 girls, 65/191 (34%) of the patients. It decreased to 33/291 (11.3%), 5/119 (4.2%), and 1/31 (3.2%) in each successive Tanner group, from 2 to 4. The area under the ROC curves was 0.709 for u_24_FSH (*P* < 0.001, Fig. 3E), 0.767 for u_24_LH (*P* < 0.001), and 0.753 for the u_24_LH/u_24_FSH ratio (*P* < 0.001). The area under the precision–recall curves (Fig. 3E) was higher for u_24_LH (0.847) than for u_24_FSH (0.772) or the u_24_LH/u_24_FSH ratio (0.765). Several hypothetical threshold values ranged in descending order of YI. The highest index thresholds were 0.035 IU/24 h for u_24_LH (sensitivity 70.9%, specificity 70.7%, YI = 0.416) and 2.0 IU/24 h for u_24_FSH (sensitivity 72.3%, specificity 62.8%, YI = 0.352). U_24_LH did not reach more than 89% sensitivity but led to a specificity higher than 90% (91.1%) by raising the threshold to 0.08 IU/24 h (Supplementary Table S2). Similarly, u_24_FSH reached a sensitivity higher than 90% (91.5%) by lowering the threshold to 1.1 IU/24 h (Supplementary Table S2). Thus, elevated u_24_LH seemed to be more interesting to identify puberty initiation (specificity) and low u_24_FSH is better to exclude it (sensitivity). It was chosen to retain and evaluate all possible combinations of the four latter above-mentioned thresholds in the validation cohort. Retrospectively, using these thresholds, the 30 GnRH tests were compared with urinary collections: 13/13 GnRH+ patients had u_24_FSH >1.1 IU/24 h and 10/13 had u_24_LH >0.08 IU/24 h, while 4/17 GnRH− patients had u_24_LH >0.08 IU/24 h (Fig. 2C). As the u_24_LH/u_24_FSH ratio did not seem to offer a better performance than u_24_LH alone, a ratio threshold was not retained.

**Table 2.** Setting cohort characteristics.

	Samples (patients)	Age (years in mean ± SD)	Age of breast development onset (years)		
			2.5th p	Median	97.5th p
All	632 (516)	8.16 ± 1.07	4.83	7.75	9.50
S1	191 (142)	7.78 ± 1.04	–		
S2	291 (242)	8.18 ± 1.03	5.15	7.80	9.67
S3	119 (107)	8.51 ± 1.03	4.70	7.50	9.25
S4	31 (25)	8.84 ± 1.06	4.70	8.00	10.20

### Validation cohort

**Figure 4.**
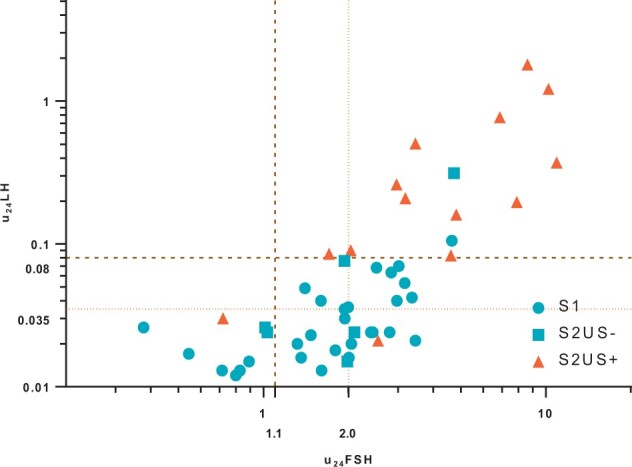
**Predictive performance of 24-h urinary gonadotropins in the validation cohort.** u_24_FSH and u_24_LH are represented in a decimal logarithmic scale. Bold dashed line stands for finally retained thresholds (1.1 IU/24 h, 0.08 IU/24 h) and slight dashed line stand for alternative thresholds evaluated (2.0 IU/24 h, 0.035 IU/24 h). FSH, follicle-stimulating hormone; LH, luteinizing hormone; S1, Breast Tanner Stage 1; S2US−, Breast Tanner stage 2 or more without ultrasonographic morphological changes; S2US+, Breast Tanner stage 2 or more with ultrasonographic morphological changes.

A total of 237 girls (375 urinary collections) were initially selected, and after exclusion, 39 patients and 49 urinary collections were finally included (Fig. 1). The age of patients at the time of inclusion were comparable in each subgroup, with mean ages between 6.44 and 7.34 years (S1, S2US−, S2US+, Supplementary Table S3). There were 15 girls who were >8 years old at the time of consultation but the clinical signs occurred before this age. Four girls had breast development at S3 Tanner stage and were merged with S2 girls. No girls presented with a S4 or S5 breast development. Among the 21 girls with Tanner stage 1 (S1), only 12 had a pelvic US and among them, two showed slight signs of an onset of morphological changes; their u_24_FSH were 1.61 and 2.4 IU/24 h, and their u_24_LH were 0.024 and <0.02 IU/24 h. Diagnostic performances of u_24_FSH and/or u_24_LH to identify S2US+ girls (predictive value, PV) were tested in all combinations of previously retained thresholds in the setting cohort (Table 3). The best positive PV was 85.7% (±9.8) combining u_24_FSH > 1.1 IU/24 h and u_24_LH > 0.08 IU/24 h and the negative PV was 94.3% (±6.5). By increasing the u_24_FSH threshold to 2.0 IU/24 h, the negative PV reached 95%, but at the expense of the positive PV (46%). In the prepubertal group, 33/35 girls had u_24_FSH or u_24_LH below 1.1 and 0.08 IU/24. Conversely, 12/14 S2US+ girls had u_24_FSH and u_24_LH above the thresholds (Fig. 4).

**Table 3.** Thresholds validation.

u_24_FSH thresholds (IU/24 h)	u_24_LH thresholds (IU/24 h)	Combined	Positive PV	Negative PV
>1.1	>0.035	OR	32.5%	88.9%
		AND	50.0%	92.0%
>1.1	>0.08	OR	32.5%	88.9%
		AND	**85.7%**	**94.3%**
>2.0	>0.035	OR	40.6%	94.1%
		AND	57.9%	90.0%
>2.0	>0.08	OR	46.4%	95.2%
		AND	84.6%	91.7%

The results in bold correspond to the combination of thresholds offering the best predictive performance that we select in this work.

Of this cohort, 27 girls were re-examined (median delay: 7 months) by the practitioners of the centre, who determined whether a progressive puberty was ongoing or not. Of the 25 girls in the prepubertal group, 17 were followed up and 15 did not have evolving puberty at the second consultation. One of the two girls in this group for whom urinary assays was found above the thresholds was still non-pubertal at follow-up. Of the 14 girls in the pubertal group (S2US+), ten were reviewed. Among these, nine with elevated urinary gonadotropin were proposed to receive puberty blockers, but in two cases the family did not choose the treatment for their child because they were close to 8 years old. Of the two out of the14 S2US+ girls for whom urinary gonadotropins were below the thresholds, one had a non-progressive breast development (premature thelarche), the other were found to have a tumour of the sex cords. Among S2US− patients, only the one with pubertal urinary gonadotropins (u_24_FSH = 4.73 IU/24 h and u_24_LH = 0.31 IU/24 h) was finally diagnosed with a slowly progressive puberty with an indication of treatment.

Overall, the combination of u_24_FSH and u_24_LH had a positive PV of 85.7% and a negative PV of 94.3% to classify prepubertal and pubertal girls in this cohort.

## Discussion

This study is the first to provide 24-h urinary gonadotropin assay data, using the CMIA reagent of Abbott Laboratories^®^, and report its usefulness as a tool for paediatrician in the diagnosis of CPP. The present results supported that u_24_FSH and u_24_LH are able to detect an increase of the gonadotropin excretion, reflecting the activation of the HPG axis. This was defined by clinical (breast Tanner stage ≥2) and US (morphological changes of internal genitalia) criteria. The choice of combined thresholds, u_24_FSH > 1.1 IU/24 h and u_24_LH > 0.08 IU/24 h, allowed the detection of CPP onset with 85.7% of positive PV and 94.3% of negative PV. The assay performed herein is a simple, non-invasive, and analytically robust technique meeting the criteria (sensitivity, specificity, and PVs) to become an alternative to GnRH test. The urinary collection can be achieved without difficulty at home, thus avoiding hospitalization, care-related stress and missing school, as well as costs and inconvenience associated with the administration of GnRH and repeated venous blood samplings.

Nocturnal FSH and LH pulses are early biological signs of central pubertal onset (Boyar *et al.*, 1972), preceding the first clinical signs (Demir *et al.*, 1996). Currently, the GnRH stimulation test is considered the gold standard to confirm central pubertal onset. However, its performance in the diagnosis of central puberty has been reported to be variable by some authors (Yazdani *et al.*, 2012; Demir *et al.*, 2016). Regarding the interpretation of the test, there are disparities of the compounds used, which are not all available in every regions of the world, and the thresholds highly vary between expert teams (Brito *et al.*, 2016). By identifying pubertal onset based on the clinic, Demir *et al.* (2016) showed that the GnRH test had a sensitivity of 59% and a specificity of 66% to distinguish S1 from S2 Tanner girls. Similarly, Yazdani *et al.* (2012) reported that the LH/FSH peak ratios exceeded 1 in only 55% of girls with central pubertal onset. In our validation cohort, we were able to use a clinical progression criterion for some patients to confirm central pubertal onset or not. Although we think this is a good indicator, further studies would be interesting to know whether this criterion is as good or better than the GnRH test. Interestingly, it was noted herein that the use of urinary gonadotropin to confirm HPG axis activation allowed us to reclass four girls as pubertal while the GnRH test was negative. Urinary gonadotropins could be able to supplement for the lack of sensitivity of the GnRH test. The response of the axis to GnRH pulses depends on sex hormone effects, involving activation of kisspeptin neurons (Cortés *et al.*, 2015; Hu *et al.*, 2022). In some situations, such as obesity, a highly emerging issue in paediatrics, a non-dynamic assessment of the rise of gonadotropin appears more appropriate than a GnRH test given the effect of leptin on kisspeptin neurons (Itriyeva, 2022). This statement should first be confirmed in a comparative study.

Several studies have proposed various protocols based on simple emptying of the bladder before bedtime and collecting in the morning, accurate notification of the time between the last emptying and collection, or standardization of the hour of collection, at one or several times (Kolby *et al.*, 2017; Shim *et al.*, 2019; Lee *et al.*, 2021; Demir *et al.*, 2023). Currently, no study has compared these methods in order to show a potential superiority. Yet the duration and intensity of gonadotropin pulsed are variable and their time of occurrence can be delayed in children with disorders of the sleep rhythm. Moreover, the studies of Wide *et al.* (2010, 2022) reported that diverse sialylated forms of glycan in physiological proportions of LH and FSH impacted their elimination clearance by basically doubling. As these forms exist in varying proportions throughout life, we think that 24-h urine collection can present interesting benefits to ensure that gonadotropin pulses are fully collected and delayed clearance are covered. The present protocol ensures that the complete circadian excretion is similarly measured for all subjects. Comparison with morning or random micturition would be of high interest. Previous studies have agreed that there is no benefit in calculating the urinary gonadotropins/urinary creatinine ratio; this does not refine correlation of values with breast Tanner stages (Zhan *et al.*, 2021), and even impairs it (Demir *et al.*, 2021). Moreover, Singh *et al.* (2015) showed in a large cohort of teenagers that an adjustment of steroid or urinary LH assays on hydration status (osmolality, specific gravity, urine creatinine) did not confer an improved relevance of the results. It was therefore chosen herein not to adjust urinary FSH and LH on creatinine.

The current study involved a first large and multicenter setting cohort to evaluate intervals and identify thresholds, and a much more restrictive single-centre validating cohort to validate the diagnostic performances of these thresholds. Good correlations of u_24_ gonadotropins with LH peaks and LH/FSH peak ratios during GnRH test, as well as with basal plasma FSH and LH levels were found. In contrast, the FSH peak after GnRH test did not correlate with any of u_24_FSH or u_24_LH. A recent study showed very similar results on randomly collected urinary samples (Lee *et al.*, 2021). Moreover, the correlation coefficients found herein were much more comparable with previous data (Demir *et al.*, 2016; Lucaccioni *et al.*, 2016; Kolby *et al.*, 2017), and higher than some others (Lee *et al.*, 2021; Zhan *et al.*, 2021).

The retained thresholds determined in the setting cohort considered the inter-individual variability of presented phenotypes, as well as that of u_24_FSH and u_24_LH in non-pubertal girls, which is clearly enlightened in this work, particularly for u_24_FSH. Yet we think that there is a possibility that the high number of paediatrician practitioners may have caused some heterogeneous over-classification of Tanner breast staging, likely to falsely downshift u_24_FSH and u_24_LH values distributions in the S2 group. This would explain the unexpected amount of u_24_LH below LLOD in S2 group, which can be explained by adipomastia or premature thelarche. Even though some patients with interfering conditions may not have been fully excluded, the number of values support the relevance of these data. We only noted one high u_24_FSH point in S1 group, which was likely to be an outlier with probable missed diagnosis of ovarian insufficiency. Two threshold values respectively for u_24_FSH and u_24_LH thresholds were chosen to be validated. The use of the YI assumes that even if CPP is a rare condition, it is not so rare in a population of girls addressed by paediatricians for clinical suspicions, and a balance between the risks of false positives or negatives is difficult to assess.

The validation cohort allowed us to confirm the usefulness of 24-h urinary gonadotropin levels. It was constructed in a sole expert centre with homogenously qualified and restricted community of paediatrician endocrinologists and radiologists, in which a bias in Tanner staging and ultrasound examination is highly unlikely. Besides, no more than three patients among 237 were excluded because of doubtful Tanner breast staging in the entire validation cohort constitution. The pre-pubertal group was thus constituted by including S2US− girls and S1 girls which, allowing a good distinction between premature thelarche and CPP, which our urinary thresholds were able to reproduce. This study showed that u_24_FSH > 1.1 IU/24 h combined with u_24_LH >0.08 IU/24 h was predictive of girls with breast development S2 or more associated with US assessment of pubertal maturation of the internal genitalia (pubertal group). Based on available progression data, one pre-pubertal S1 girl had elevated urinary gonadotropins but was still pre-pubertal at follow-up. The interpretation of urinary assays was very consistent with clinical conclusion, as only S2 (or more) girls for whom urinary gonadotropin levels were greater than the thresholds were diagnosed with CPP and received puberty blockers. Two S1 girls showed slight signs of an onset of morphological evolution (S1US+), and although their u_24_FSH were >1.1 IU/24 h, u_24_LH was quite low (<0.035 IU/24 h or undetectable). It is difficult to conclude whether these two girls were at a very early step of puberty initiation, in which case, internal genitalia morphologic changes would be visible before breast development. Without a detectable rise of urinary LH, this would be congruent with the limited lack of sensitivity of the u_24_LH threshold. Nevertheless, it is possible that some girls having u_24_LH comprised between 0.035 and 0.08 IU/24 h may be in a ‘middle-zone’ of awakening the HPG axis prior to any clinical or US signs. This should be elucidated in a prospective study. On the other hand, three S2US+ had only slightly elevated u_24_LH higher than 0.08 (0.083, 0.085, 0.09 IU/24 h). According to coefficient of variation at their raw levels (IU/l), the repeat of the assay would presumably have not classified them differently. Yet the inherent limit of a threshold must be considered, and a control collection should be advised in case of any borderline results. No patients had u_24_FSH < 1.1 IU/24 h with u_24_LH > 0.08 IU/24 h, which appears to be a paradoxical hypothetical situation, that may evoke pre-analytical or analytical errors.

The present results lead to support the idea that 24-h urinary gonadotropin measurements have their place in the diagnosis of CPP onset, regarding the predictive performances presented herein. Studies from last decade reporting data on FMV urines or random urines have shown good usefulness, with sensitivity from 65% to 92% and specificity from 63% to 100% (Demir *et al.*, 2021; Zhan *et al.*, 2021). A meta-analysis from Xu *et al.* (2022) that retained six studies, in spite of heterogeneous collecting protocols (collection protocols, sample treatment prior to analysis) and analytical techniques, revealed an overall sensitivity of 79% and specificity of 84%. Comparisons of diagnostic thresholds from different studies must consider these pre-analytical and assay method differences. It must, however, be considered that the proposed and validated thresholds from this study can only apply to an Abbott Laboratories^®^ CMIA assay performed directly on non-extracted 24-h urine; measurement without extraction using other reagents must be evaluated due to a likely high heterogeneity. This automated immunoassay kit for gonadotropins in urine is highly correlated with RIA techniques (Supplementary Fig. S1), which had demonstrated their value in clinical practice in the past (Morel *et al.*, 1985).

However, this study has some limitations. First, related to its retrospective design, further prospective follow-up studies should be performed to describe the chronology of the increase of 24-h urinary gonadotropins and that of clinical pubertal development. Long-term clinical follow-up data on pubertal progression was only partly available for patients in both the prepubertal and the pubertal groups (27/39). Although there was a good association between urinary gonadotropin levels and the decision to treat or clinical progression in these patients, the retrospective nature of the study means that we cannot rule out the possibility that urinary gonadotropin levels may have influenced management. Also, there is a margin of error due to the inherent uncertainty of clinical assessment of pubertal onset. These limitations specific to clinical examination linked to the number of practitioners must be kept in mind, particularly in the setting cohort, in which the geographical scope of inclusion was wide. Finally, urine sampling, although requiring only basic equipment, is sometimes subject to omission or involuntary urine loss by children, which can compromise the completeness of the collection. This is an easy sampling procedure, but imposes certain pre-analytical requirements. Assays can be easily repeated on new 24-h collections. The authors also point out that this study was made possible by the applicability of the Abbott assay kit to urine samples, and that this assay is not available to all centres treating these patients. As another strong point, this setting cohort was based on the inclusion of a large number of girls with no breast development (142 S1 girls with 191 urines); this provided us an original control group of girls with clinically non-initiated central puberty. One of the strengths of the validation cohort is that it was constructed using US criteria focused on the uterus and on the ovaries, and performed at the same time that the urinary collection. Moreover, the large number of values in the setting cohort is a strong argument for the reliability of the intervals.

In conclusion, the present results provide evidence of the reliability of 24-h urinary gonadotropins in the diagnosis of CPP in girls, as our method performs at least as well as the GnRH test and is well predictive of clinical and radiological pubertal onset. The herein presented u_24_FSH and u_24_LH thresholds must be used in further prospective studies aiming to compare with the GnRH test for diagnosis and treatment monitoring, as well as with FMV or random collection of urine samples.

## Supplementary Material

deae055_Supplementary_Figure_S1

deae055_Supplementary_Table_S1

deae055_Supplementary_Table_S2

deae055_Supplementary_Table_S3

## Data Availability

Data are available on request.
