# Supplementary material for: Urinary gonadotropin assay on 24-h collections as a tool to detect early central puberty onset in girls: determination of predictive thresholds
Source: Hum Reprod. 2024 Mar 21;39(5):1003–12. doi: 10.1093/humrep/deae055 (PMC11063551; doi:10.1093/humrep/deae055)
Supplement: deae055_Supplementary_Figure_S1 [file deae055_supplementary_figure_s1.pdf]

Human Reproduction, 2024, 1–1
https://doi.org/10.1093/humrep/deae055
Supplementary Data

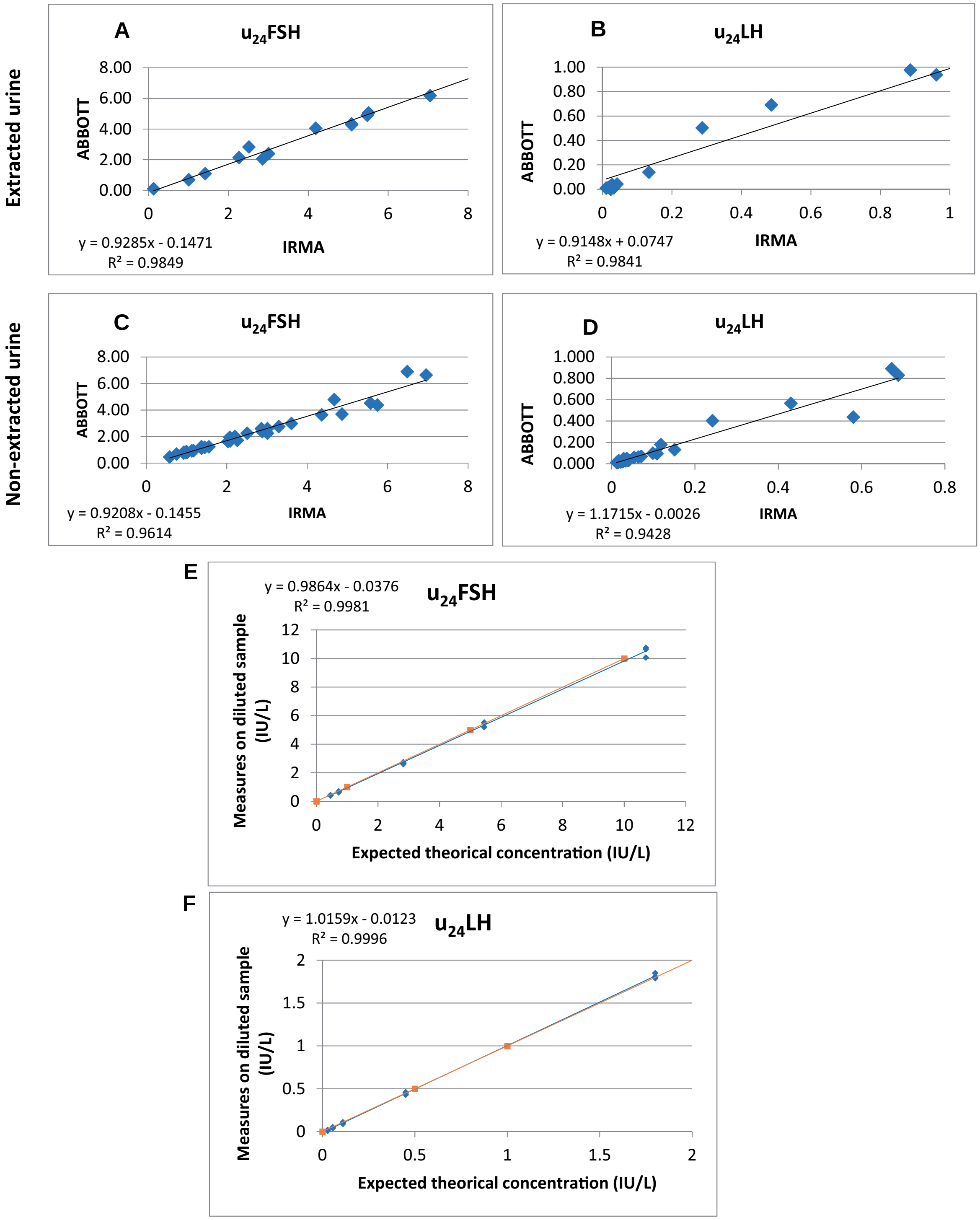

Supplementary Figure S1. Analytical validation of the urinary gonadotropins method. (**A** and **B**) Correlation between the IRMA method and chemiluminescent automatized method from Abbott Laboratories on extracted urinary samples. (**C** and **D**) Correlation between the IRMA method and chemiluminescent automatized method from Abbott Laboratories on non-extracted urinary samples. (**E** and **F**) One sample containing 10.7 IU/l of FSH was diluted to ½, ¼, 1/20, and 1/40, and one sample containing 1.8 IU/l for LH, diluted to ½, ¼, 1/16, 1/32, and 1/64, were measured to assess the linearity of the method. Orange points stand for the equality line ($y = x$). FSH, follicle-stimulating hormone; IRMA, immunoradiometric assay; LH, luteinizing hormone.
