# Supplementary material for: Urinary gonadotropin assay on 24-h collections as a tool to detect early central puberty onset in girls: determination of predictive thresholds
Source: Hum Reprod. 2024 Mar 21;39(5):1003–12. doi: 10.1093/humrep/deae055 (PMC11063551; doi:10.1093/humrep/deae055)
Supplement: deae055_Supplementary_Table_S1 [file deae055_supplementary_table_s1.pdf]

Human Reproduction, 2024, 1–1
https://doi.org/10.1093/humrep/deae055
Supplementary Data

Supplementary Table S1. Sensitivity of the ECLIA reagent from Abbott Laboratories® in urine samples.

		Number of repeated measurements	Mean (IU/l)	CV (%)
FSH	1	15	0.27	4.15
	2	15	1.6	4.1
	3	15	12.8	3
LH	1	15	0.016	39%
	2	15	0.062	10.2%
	3	15	0.38	6.5
	4	15	1.68	2.6

Repeated measures (repeatability) on different concentrations levels in urine samples are shown in the table.
ECLIA, electrochemiluminescent immunoassay; FSH, follicle-stimulating hormone; LH, luteinizing hormone.
