# Supplementary material for: Urinary gonadotropin assay on 24-h collections as a tool to detect early central puberty onset in girls: determination of predictive thresholds
Source: Hum Reprod. 2024 Mar 21;39(5):1003–12. doi: 10.1093/humrep/deae055 (PMC11063551; doi:10.1093/humrep/deae055)
Supplement: deae055_Supplementary_Table_S2 [file deae055_supplementary_table_s2.pdf]

Human Reproduction, 2024, 1–1
https://doi.org/10.1093/humrep/deae055
Supplementary Data

Supplementary Table S2. Different thresholds performances in the setting cohort.

u_{24}FSH thresholds (IU/24 h)	Sensitivity	Specificity	Youden index	u_{24}LH thresholds (IU/24 h)	Sensitivity	Specificity	Youden index
>0.78	95.0	12.0	0.071	>0.020	80.8	54.4	0.353
>0.94	92.9	19.4	0.123	>0.024	76.6	61.2	0.382
>1.1	91.5	24.6	0.161	>0.035	70.9	70.7	0.416
>1.3	87.2	34.5	0.218	>0.050	57.0	81.1	0.382
>1.7	79.8	48.7	0.285	>0.067	50	88.4	0.385
>2.0	72.3	62.8	0.352	>0.080	46.1	91.1	0.372
>2.5	64.2	64.9	0.291	>0.101	38.3	93.7	0.320
>3.7	32.6	90.0	0.227	>0.144	30.1	97.9	0.286

u_{24}FSH, 24-h urinary follicle-stimulating hormone; u_{24}LH, 24-h urinary luteinizing hormone.
