# Supplementary material for: Urinary gonadotropin assay on 24-h collections as a tool to detect early central puberty onset in girls: determination of predictive thresholds
Source: Hum Reprod. 2024 Mar 21;39(5):1003–12. doi: 10.1093/humrep/deae055 (PMC11063551; doi:10.1093/humrep/deae055)
Supplement: deae055_Supplementary_Table_S3 [file deae055_supplementary_table_s3.pdf]

OXFORD
human reproduction

Human Reproduction, 2024, 1–1
https://doi.org/10.1093/humrep/deae055
Supplementary Data

Supplementary Table S3. Validation cohort characteristics.

	Samples (patients)	Age (years in m±SD)
All	49 (39)	7.21 ± 1.15
S1	29 (21)	7.34 ± 0.82
S2US−	6 (6)	6.44 ± 2.00
S2US+	14 (14)	7.27 ± 1.24

S1, Tanner Stage 1; S2US−, Tanner Stage 2 with ultrasonic (US) morphological changes; S2US+, Tanner Stage 2 with US morphological changes.
